# Network organizations of general practitioners: antecedents of formation and consequences of participation

**DOI:** 10.1186/1472-6963-10-118

**Published:** 2010-05-11

**Authors:** Giovanni Fattore, Domenico Salvatore

**Affiliations:** 1 Department of Public Management and Institutional Analysis and CERGAS, Università Bocconi, via Roentgen 1, 20136, Milan, Italy; 2 Department of Management, Università Parthenope, Via Medina 49, 80133 Naples, Italy; 3 Fondazione SDN, Via Gianturco 113; 80143 Naples, Italy

## Abstract

**Background:**

Network forms of organization are increasingly popular in primary care. At the end of the 1990s General Practitioners (GPs) in Italy were given the opportunity to adopt network forms of organization with the aim of improving the quality of their services. However factors affecting GPs' choices to join a network and the consequences of network membership have not been evaluated.

**Methods:**

Administrative data of a Local Health Authority in Central Italy were analyzed using statistical methods at individual and dyadic levels of analysis.

**Results:**

Homophily factors seem to influence a GP's choice of network. The consequences of network membership on GP performances seem very limited.

**Conclusions:**

When considering to foster the diffusion of network organizational forms in health care creating a network structure, like that of Italian GPs, is not sufficient. Other features of the implementation phase, work organization and human resource management should also be considered.

## Background

In organization theory, the idea that a network is a form of organization of activities like hierarchy (i.e., a company or a public organization) or market, found legitimacy and popularity during the first half of 1990 [1-3]. The network form of governance of activities which is characterized by "reciprocal patterns of communication and exchange" [1] has been described as a hybrid form of organizations of activities offering some advantages over both hierarchies and markets. During the same period, national and regional governments deeply reformed the Italian National Health Service (INHS), and by the end of the decade a network form of organization of general practice was institutionalized in various regions [4,5]. Those networks of general practitioners (henceforth GPs), termed "forme associative" in Italian, have been presented by politicians, administrators and GP unions as the solution to many of the problems affecting the organization of GPs in Italy. This is because they create an organizational structure for otherwise isolated professionals and facilitate knowledge exchange while maintaining the administrative autonomy that Italian GPs had always enjoyed. In this article we will describe the structure of GP networks in Italy and report evidence of the antecedents of their formation and their consequences on GPs' behaviour in one Italian province.

Networks offer an intermediate arrangement between the flexibility offered by markets and the stability of hierarchies. In a network organization actors enjoy an independence similar to that which they enjoy in markets and have incentives in order to increase efficiency and easy-to-observe quality similar to those present in markets. These incentives also act to increase GPs' flexibility to experiment and adopt innovations that may be useful in their specific context. In addition, when compared to market exchanges, network arrangements frame repeated interactions and create greater communication between GPs, that in turn facilitate knowledge sharing and the diffusion of innovations. Furthermore, better circulation of information generates greater opportunities to evaluate difficult-to-observe quality and increases the cost of behaving opportunistically.

In a network organization actors also enjoy stability of relationships similar to that present in hierarchies. Actors can develop trust and are able to make organization-specific investments such as specializing in some activities which would have lower value if the GP was independent or moved to another organization. But when compared to hierarchy, networks allow greater efficiency and flexibility in knowledge-intensive activities. This is mainly because networks better control opportunistic behaviour through norms of reciprocity and reputation concerns and therefore avoid the need of direct supervision or of ex-ante specification of processes characterizing bureaucratic control.

At the individual level of analysis, social network theorists have focused on the behavioural, perceptual, and attitudinal consequences [6] of the social relationships in which actors are embedded [7,8]. Those relationships are the ties linking nodes of the networks and are also said to be the social capital of actors [9,10]. Ties influence actors' behaviours through four mechanisms: network closure determines the social control typical of small and closed communities; brokerage determines the power and informational advantage of actors bridging other actors otherwise disconnected; contagion determines the adoption of a characteristic of connected actors; and, prominence which favours the adoption of an actor's characteristic who is central to the network.

One variable that many social network theorists have been concerned with is the redundancy of connections, which is the degree to which an actor's ego network is made by contacts that are already connected to each other. Being connected to a highly redundant network implies having access to fewer resources than someone connected to a non-redundant network with the same amount of ties. Non redundant contacts create social capital through the brokerage mechanism [11].

## Methods

### Context

The INHS was established in 1978 and was modelled after the British NHS. It is a mainly public system financed through general taxation [12]. From an organizational viewpoint the INHS divides the Italian territory into 180 Local Health Authorities (LHAs) each responsible for the provision of health services to its population.

GPs are primary care physicians working for LHAs as independent contractors and act as gatekeepers of higher levels of care. In 2005 there were 47,022 GPs in Italy costing about 5 billion Euros, or 6% of public healthcare expenditure [13]. Traditionally, Italian GPs work in solo practices without any auxiliary staff or any formal linkages with other GPs. Their status as independent but exclusive contractors with a LHA is coupled with a financing system based on capitation: GPs are paid on the basis of the number of patients on their list [14]. The freedom of patients to choose the list they are on and to switch GP at any moment induces a form of competition among GPs to maintain current and acquire new patients.

Since the INHS inception in 1978, various initiatives have slowly but constantly shifted resources and responsibilities to non-hospital settings. The number of hospital beds in Italy decreased by 28% from 1995 to 2005 [15]. This process of de-hospitalization of care has increased the workload and the responsibilities of GPs, as they are a pivotal part of community care, given their gatekeeping role and their direct knowledge of patients. Nonetheless, Italian health reforms have not changed the role nor the organization of the GPs' activities.

The diffusion of network forms of organization eventually reached the organization of Italian general practitioners through their definition in the national contract agreement. This represented the main, if not the only, major organizational innovation of GPs' activity since the inception of the INHS in 1978. GP networks were and still are presented as the solution to many of the problems affecting the organization of primary care in Italy as they create an organizational structure and facilitate knowledge exchange, while maintaining GPs' administrative autonomy.

### General practitioners networks

The contract according to which GPs provide services on the behalf of the INHS to citizens enrolled in their list is negotiated every three years by the GP unions with a governmental agency. This contract is the main document regulating the relationship between GPs and the INHS. Additionally, integrative contracts may be signed by unions and INHS organizations at a regional or local level. Through the national contract organizational innovations, such as the GP networks, are introduced into the system. The first national contract mentioning the idea that organizational forms creating some collaboration among GPs could be negotiated at a local level was signed in 1996. However, it was only when the contract was renewed in 2000 that the regulation of GP networks was detailed.

In the national contract in place at the time of the study, GPs willing to undertake some form of collaboration in the provision of health services to their patients can choose among three forms. They are named association ("medicina in associazione"), net ("medicina in rete"), and group ("medicina di gruppo") and each implies a different strength of the collaboration among GPs (Table 1). By simply joining one of these typified forms GPs have the right to additional compensation from the INHS which tops an 18% increase over the base GP salary. None of these GP networks are legal entities and every contractual relationship is between the INHS and the individual GP. In any of these three forms GPs have to coordinate their office hours to remain open till 7 pm on weekdays and commit to share guidelines and meet to discuss and improve their work. Each patient is listed with a GP but when the GP is unavailable the patient may be examined by any other GP from the same network. The freedom of patients to leave their GP and to enrol in the list of another in the same network is limited in order to mitigate the concern GPs may have that, by collaborating they could lose patients to their colleagues. In the case of networks of intermediate intensity, nets, GPs are also required to share an electronic database of their patients. While in associations or nets GPs can continue working in their own offices, but the most intense form, the group, requires GPs to share clinics and, therefore they can share investments in medical equipment and employ nursing or administrative staff. In the LHA analyzed in this study, an LHA-employed specialist performed ultrasound procedures once a week in each of the group clinics. Although at the time of the study the LHA was thinking about asking GPs in groups to increase the sharing of clinics with specialists, the three network arrangements are essentially uniprofessional organizational forms.

**Table 1:** Features of the three forms of collaborative initiatives typified in the national labour contract of GPs (adapted from Brunello, 2001[30]).

	Associations	Nets	Groups
Number of members	3 to 10	3 to 10	3 to 8

Minimum number of office hours during weekdays	6	6	6

Shared electronic patient records	No	Yes	Yes

Shared ambulatory	No	No	Yes

Additional income for GP for each enrolled patient	2.58 € (7% increase)	4.70 € (12% increase)	7 € (18% increase)

The basic idea behind these networks is that organizational and professional development in primary care requires collaboration between GPs and the sharing of resources and knowledge. This means networks can improve service delivery (longer office hours, continuity of care, wider range of services). As of 2004, 59% of Italian GPs have entered a type of network, 22% of which joined a group, the more intense organizational form [16]. GP networks are indeed networks of peers in which each individual GP is at liberty to choose to join colleagues he/she prefers. Contrary to experiences in other countries with primary care organizations, GP networks in Italy are organizational arrangements in which every member is a GP and specialists are not involved. Although most groups (the more intense and less adopted form) employ a part-time nurse or an administrative assistant, associations, nets, and groups are better understood as uni-professional networks. Members of each network are free to choose their governance structure, and given that these organizational forms are small networks of peers which GPs can leave whenever they wish, it is very likely that decisions are made by unanimous agreement. Moreover, the sharing of guidelines and meetings to discuss and improve professional work, two main requirements of GP networks, are vaguely defined and very difficult to verify by the LHA.

### Data

Data used for the analysis were provided by the Piacenza LHA in Emila-Romagna, a region in the central part of the country. This LHA is responsible for an area with approximately 280.000 inhabitants and comprises of a town of 100.000. The LHA administrative information systems contain a large data-set useful to investigate both antecedents and consequences of participation in GP networks. In 2006, the year analyzed in this study, there were 1.23 GPs for every 1,000 residents in the LHA; 69% of GPs had entered a type of network and 16% of them joined a group.

Some of the analyses reported here were performed at the individual level of analysis (i.e. the individual GP) and some were performed at the dyad-level of analysis (i.e., the unit of observation is the pair of GPs). We have therefore individual-level data on 223 GPs which, at the dyad-level of analysis became 49,506 pairs.

The Piacenza LHA did not introduce at the local-level significant additions to the national-level contract in place at the time of the study. Therefore, although the LHA periodically sends to its GPs the statistics on most indicators used in this study, there was not a formal budgeting process recognizing incentives to GPs who meet LHA's target. Table 2 reports descriptive statistics for all variables.

**Table 2:** Means, standard deviations and correlations of the variables employed in the analyses.

	Mean	Std.Dev	1	2	3	4	5	6	7	8	9	10	11	12	13	14	15	16	17	18	19	20	21
1. Gender^1^	0.21																						

2. DistrictA	0.27		0.21																				

3. DistrictB	0.06		0.06	-0.15																			

4. DistrictC	0.19		-0.05	-0.29	-0.12																		

5. Years since graduation	25.10	6.85	-0.36	-0.10	-0.03	0.02																	

6. Number of patients	1111.22	463.01	-0.33	0.01	-0.08	0.02	0.50																

7. Risk AdjInd.	1.8	0.26	-0.09	0.05	0.45	-0.01	0.17	0.23															

8. Specialized	0.65		-0.03	0.06	-0.02	0.06	0.16	0.00	0.05														

9. In any network	0.67		-0.06	-0.10	-0.03	-0.12	0.28	0.43	0.13	-0.04													

10. Association	0.3		0.08	0.04	0.01	-0.20	0.06	0.09	0.05	0.02	0.46												

11. Net	0.3		-0.13	-0.31	0.00	0.15	0.21	0.29	0.08	-0.05	0.47	-0.43											

12. Group	0.07		-0.01	0.32	-0.07	-0.13	0.03	0.11	0.00	-0.03	0.19	-0.18	-0.18										

13. Pharma	115.45	82.29	0.15	0.02	0.24	-0.02	-0.09	-0.11	0.34	0.07	0.06	-0.05	0.10	0.03									

14. Home Care	7.23	6.93	-0.04	0.08	0.18	0.27	0.05	0.18	0.20	0.06	-0.04	-0.08	0.06	-0.04	-0.01								

15. Multi-prof. home care	13.65	12.49	-0.05	0.02	-0.01	-0.04	0.03	0.27	0.19	0.08	0.07	0.06	0.03	-0.03	0.02	0.22							

16. Printed Prescriptions	0.7	0.36	-0.07	0.02	-0.13	-0.21	0.15	0.27	-0.10	0.01	0.43	0.09	0.28	0.14	0.00	0.02	-0.01						

17. White Codes day	7.09	5.04	-0.01	0.13	-0.15	0.05	0.13	0.42	-0.10	0.00	0.11	0.01	0.12	-0.04	0.02	0.15	0.12	0.1					

18. White Codes Total	37.85	20.56	-0.09	0.13	-0.15	0.15	0.26	0.60	-0.01	0.02	0.12	0.00	0.16	-0.09	0.06	0.16	0.13	0.12	0.79				

19. Preventable re-admissions	5.01	3.59	-0.25	-0.10	0.11	-0.11	0.29	0.54	0.27	-0.08	0.20	0.05	0.20	-0.08	-0.05	0.06	0.31	0.07	0.2	0.36			

20. Screening Paptest	0.44	0.13	0.12	0.44	-0.16	-0.19	0.18	0.15	0.17	-0.02	0.11	0.04	0.00	0.12	0.07	-0.01	-0.02	0.03	0.12	0.13	0.03		

21. Screening (average)	0.55	0.16	0.05	0.21	-0.21	-0.21	0.24	0.35	0.11	0.06	0.10	-0.02	0.11	0.01	-0.09	-0.03	0.12	0.2	0.23	0.29	0.24	0.68	

22. Performance Index	106.75	23.15	-0.04	-0.06	0.02	0.20	0.05	0.25	0.10	-0.09	0.01	0.01	0.05	-0.08	0.26	-0.24	-0.15	-0.24	0.47	0.53	0.40	-0.1	-0.17

^1 ^Male = 0; Female = 1; See text for further explanation of variables

### Variables

#### Participation in a network

Based on administrative data, we created affiliation matrices linking each GP to all possible networks. The LHA keeps a record of which network each GP has joined for its administrative purposes. In the following analyses we used this binary variable both as independent and dependent variables. For the analyses conducted at the dyadic level of analysis this variable becomes "participation in the same network" and is coded 1 if both GPs are members of the same network and 0 if they are members of different networks or are not members of any network. Indeed, a connection between two GPs exists when they are members of the same network. Such type data are named in social network analysis "affiliation networks" [17].

#### GP performance dimensions

GPs' activities are complex and it is impossible to find any single variable which may satisfactorily represent their performance. Using the data collected by the LHA, we reported the analyses at the individual and dyadic levels on the consequences of participation in GP networks using ten dependent variables. Nine of them capture specific aspects of their performance, and one is a performance index that summarizes them. The first variable "Pharma", is per-capita pharmaceutical expenditure of the patients enrolled with a GP. With the exception of some particular categories of drugs, patients in Italy need GP prescriptions to obtain drugs under the INHS. GPs are therefore gatekeepers of their patients' pharmaceutical expenditure and they are under pressure to keep it as low as possible in order to keep INHS expenditure at a minimum. The second, "Planned Home Care" measures how many of the GP's patients are examined regularly at home; home care is specifically encouraged by LHAs as it is assumed to reduce inappropriate hospitalization and increase the quality of care for patients. For the same reason, GPs may coordinate a group of other professionals (for example nurses, therapists and specialists) to regularly examine the patient; the number of patients benefiting from this activity is measured by the "Integrated home care" variable. Planned home care differs from integrated home care because in the latter the GP not only visits the patient home but also coordinates the accesses to the patient's home of a team of health professionals and specialists. The "Preventable re-admissions" variable measures the number of patients who are re-admitted to hospital for conditions that could have been managed effectively by the GP. This measure detects a lack of adequate continuity of care after the patient's discharge. "Printed prescription" is the percentage of prescriptions that have been printed by the GP using a computer (and thus not handwritten). The printing of prescriptions is encouraged by the LHA as it guarantees that the GP keeps an electronic archive, increase safety, and eases part of the subsequent administrative work. "Screening Paptest" is the fraction of targeted patients by the paptest screening program managed by the LHA, who have joined the program; "Screening (average)" is the average participation rate of the patients to the three screening programs that was in operation in the LHA during 2006: pap-test, mammogram, and colon-rectal. GPs are assumed to play a role in promoting health to their patients and inform them about the advantages of the screening programs. Two indicators refer to patients' access to Emergency department and are intended to capture the role that GPs may have played in its inappropriate use. "White Codes day" is the number of patients' accesses to emergency departments that resulted in a white code (lowest level of priority) during GPs' working hours; "White Codes Total" is the same indicator applied to any access, thus including those at night or during week-ends. GPs are assumed to be able to manage most of the non-urgent health issues and white codes can be partially attributed to the quality of GPs' work. To try to portray a single measure of the GPs' performance we ranked each GP according to these nine performance dimensions and thus we created a tenth variable "Performance Index". GPs' performance is a multidimensional concept which should consider aspects related to quality of care, organization, patient satisfaction, equity and cost containment. The "performace index" used in this study is not a comprehensive indicator of GP performance, but is simply the average rank of a GP alongside the nine dependent variables previously presented.

All these variables have been calculated on a per-patient basis, that is dividing the dependent variable by the number of patients enrolled in each GP list. For the analyses at the dyadic level each variable becomes the difference in the value of the variable of the first and the second GP in the dyad.

#### Control Variables

The control variables used in the models include gender, the years since the medical degree, a dummy variable coded 1 if the GP has any kind of specialization other than the medical degree, the number of patients that are in the GP's list; an age standardization index depending on the age of the enlisted patients which is used by the LHA to weigh the number of patients against their expected total healthcare costs. Finally we used three dummy variables to identify the district in which the GP operates. Districts are the organizational units of the LHA to which GPs refer administratively and are supposed to provide the same primary care services to their residents. One of the four districts provides services to the city of Piacenza and it is used as the reference category in the statistical analysis. The other three offer services to countryside or mountain areas. Districts provide similar services and are organized in similar ways, nonetheless social and geographical characteristics of the areas differ; moreover districts have a different management team and therefore we controlled for this variable in the analyses and reported it in the models for which it played a role.

#### Participation in a network

Based on administrative data, we created affiliation matrices linking each GP to all possible networks. The LHA keeps a record of which network each GP has joined for its administrative purposes. In the following analyses we used this binary variable both as independent and dependent variables. For the analyses conducted at the dyadic level of analysis this variable becomes "participation in the same network" and is coded 1 if both GPs are members of the same network and 0 if they are members of different networks or are not members of any network. Indeed, a connection between two GPs exists when they are members of the same network. Such type data are named in social network analysis "affiliation networks" [17]. In the LHA we studied, every network was participated by GPs in the same district.

### Analysis

We used these data to study both the factors affecting the choice to enter a GP network and the consequences of being a member. In both cases we performed the analysis at the individual level and at the dyad level. By "dyad" we mean each of all the possible pairs of GPs in the sample. At the dyadic level of analysis values of the variables for each dyadic observation have been calculated as the difference of the value of the first GP and the value of the second in the case of continuous variables, or by the presence of a characteristic for both GPs in the case of binary variables.

Along with standard linear and logistic regression we used two statistical procedures typical of social network analysis studies: exponential random graph model (ERGM) and quadratic assignment procedure (QAP).

ERGM, also called the p* class of models, which is a probability model for networks on a given set of actors allowing generalization beyond the restrictive dyadic independence assumption [18,19]. The analysis was performed using the software SIENA [20].

QAP is a multiple regression technique able to regress a valued matrix on other matrixes and does not assume independence of observations [21]. MR-QAP was performed using the UCINET software package [22].

## Results

### Factors affecting the choice to enter a GP network

Table 3 shows the results of a logistic regression for which the dependant variable was the participation of a GP in any of the three GP networks. Among the GPs of the studied LHA, the number of patients has a small but highly significant effect and being female makes it more likely to join a network (p < 0.1).

**Table 3:** Results of a Logistic Regression Model for the membership in a collaboration initiative (-2 Log likelihood = 232.720; Cox & Snell R Square = 0.220; Nagelkerke R2 = 0.303).

Variable	Beta (standard errors)
Gender	0.812 (0.440)*

Years since graduation	0.032 (0.023)

Specialized	-0.453 (0.343)

Number of patients	0.002 (000)**

Constant	-2,115 (0.614)**

* p < 0.10; ** p < 0.05.

In Table 4 we present an analysis at the dyadic level which investigates factors predicting the presence of a connection between two GPs. The analysis uses an ERGM procedure. The dependant variable is the co-membership of two GPs in the same collaboration initiative (the level of analysis is thus, dyadic) and the independent variables are: being of the same gender, the difference in the number of years since graduation from medical school, the GPs' specialization (if any), and the similarity in the number of patients enrolled. Two variables are significant: years since graduation and number of enrolled patients. This suggests that GPs tend to join a GP network with colleagues of their own generation and with colleagues that have a similar number of patients (therefore a similar need of common resources).

**Table 4:** Results of an exponential random graph regression model for the presence of any connection between two actors (n = 223).

Variable	Estimates (standard errors)
Same Gender	-0.0639 (0.1490)

Difference in years since graduation	1.5726 (0.5109)**

Both having a medical specialisation	-0.0606 (0.1226)

Difference in the number of patients	1.7080 (0.3132)**

** p < 0.05

### Consequences of participation to GP networks

To study the consequences of participating in any GP network, we used an ordinary least square (OLS) regression. Table 5 reports 10 regression models, where the same set of regressors are tested and 10 different dependent variables are used (9 specific measures and an overall performance index). The two variables of interest are "in any network" representing the participation of the GP in any of the three forms of network, and "in group" representing the participation in the network implying the most intense collaboration. As shown in Table 5 being in a network has a positive and significant effect only on the percentage of prescriptions printed using a computer. Being in a group has a significant effect only in reducing the number of patients going to a hospital emergency department for a condition classified as a white code.

**Table 5:** Standardized regression coefficients (p-value) of linear regression models with individual GPs performance indicators as dependent variables.

	Pharma	Planned Home Care	Integrated home care	Preventable re-admissions	Printed Prescriptions	Screening Paptest	Screening (average)	White Codes day	White Codes Total	Performance Index
Intercept	0.00 (0.94)	0.09 (0.20)	0.01 (0.88)	0.00 (0.95)	0.02 (0.77)	-0.02 (0.69)	0.00 (0.97)	0.00 (0.97)	0.01 (0.92)	-0.01 (0.81)

Gender	0.11 (0.11)	0.07 (0.38)	0.04 (0.59)	-0.01 (0.95)	-0.01 (0.93)	0.15* (0.02)	0.22** (0.00)	0.18* (0.01)	0.23** (0.00)	0.11 (0.13)

DistrictA	-0.02 (0.80)	0.09 (0.28)	-0.17* (0.04)	-0.17* (0.04)	-0.02 (0.81)	0.39** (0.00)	0.06 (0.39)	0.06 (0.48)	0.06 (0.38)	-0.01 (0.89)

DistrictB	0.05 (0.54)	0.10 (0.26)	-0.17* (0.05)	-0.08 (0.30)	-0.07 (0.34)	-0.18** (0.01)	-0.30** (0.00)	-0.05 (0.51)	-0.16* (0.03)	-0.01 (0.89)

DistrictC	0.02 (0.81)	0.15 (0.06)	-0.14 (0.07)	-0.21** (0.01)	-0.14* (0.04)	-0.14* (0.02)	-0.24** (0.00)	0.06 (0.38)	0.18** (0.01)	0.20** (0.00)

Years since graduation	-0.03 (0.68)	-0.07 (0.44)	-0.02 (0.86)	-0.01 (0.92)	0.01 (0.94)	0.14* (0.05)	0.16* (0.03)	0.04 (0.62)	0.05 (0.56)	-0.09 (0.26)

Number of patients	-0.2* (0.02)	-0.32** (0.00)	-0.17 (0.08)	-0.05 (0.56)	0.12 (0.15)	0.11 (0.15)	0.33** (0.00)	-0.03 (0.71)	-0.09 (0.3)	-0.08 (0.33)

Age St. Ind.	0.38** (0.00)	0.11 (0.28)	0.30** (0.00)	0.21* (0.01)	-0.12 (0.09)	0.14* (0.03)	0.18* (0.01)	-0.26** (0.00)	-0.18* (0.02)	-0.04 (0.65)

Specialized	0.07 (0.28)	-0.01 (0.87)	0.11 (0.11)	-0.07 (0.34)	0.05 (0.4)	-0.08 (0.16)	0.04 (0.54)	-0.03 (0.6)	0.00 (0.99)	-0.08 (0.21)

In any network	0.11 (0.14)	-0.15 (0.07)	-0.13 (0.10)	-0.09 (0.26)	0.37** (0.00)	0.04 (0.56)	-0.12 (0.09)	0.01 (0.94)	-0.11 (0.12)	-0.10 (0.19)

In group	0.04 (0.56)	-0.04 (0.57)	0.03 (0.66)	-0.06 (0.44)	0.04 (0.53)	-0.07 (0.27)	-0.08 (0.24)	-0.08 (0.28)	-0.13* (0.05)	-0.05 (0.48)

R2	0.19	0.21	0.11	0.09	0.24	0.34	0.30	0.14	0.24	0.14

Adj R2	0.15	0.16	0.06	0.04	0.21	0.31	0.27	0.09	0.21	0.10

* p < 0.05; ** p < 0.01

To analyze the consequences of participation in a GP network at the dyadic level we ran a series of QAP regressions using as dependant variables the similarity in each of the ten performance dimensions of all the possible dyads of GPs in the sample (Table 6). Being in the same network significantly makes two GPs similar in the percentage of prescriptions they print with a computer, suggesting that some form of contagion or of social control takes place inside networks. Being in the same network also makes two GPs significantly more different in the number of the patients they examine at home, suggesting that some form of division of labour occurs. Considering all the dimensions of GP performance for which we had data, overall, the standardized effect on the similarity of performance of GPs in shared networks are very small and generally non-significant.

**Table 6:** Standardized regression coefficients of quadratic assignment procedure (QAP) regression models with the similarity of any two GPs performance indicators as dependent variables.

	Dependent variables									
	Δ	Δ	Δ	Δ	Δ	Δ	Δ	Δ	Δ	Δ
	Pharmaceutical expenditure	Planned Home care	Integrated home care	Printed prescriptions	Day-time white codes	Total white codes	Preventable readmissions	Screening paptest	Screening average	Performance index
Intercept	0.00	0.00	0.00	0.00	0.00	0.00	0.00	0.00	0.00	0.00

Same Gender	0.00	0.00	0.00	0.00	0.00	0.00	0.00	0.00	0.00	0.00

Grad YY diff	-0.03	-0.07	0.00	-0.01	-0.01	-0.03	-0.05	0.16*	0.08	-0.11

Number patients diff	-0.09	-0.20**	-0.14*	0.30**	-0.13	-0.2**	-0.02	0.08	0.30**	-0.19**

Age st. diff	-0.11*	-0.13*	0.08	0.20**	0.01	-0.06	0.17**	-0.19**	0.06	-0.11*

Both specialized or not	0.00	0.00	0.00	0.00	0.00	0.00	0.00	0.00	0.00	0.00

Same any dist	0.00	0.00*	0.00	0.00*	0.00	0.00	0.00	0.00	0.00	0.00

Both in any network	0.00	0.00*	0.00	0.00*	0.00	0.00	0.00	0.00	0.00	0.00

Both in any group	0.00	0.00*	0.00	0.00*	0.00	0.00	0.00	0.00	0.00	0.00

Both same network	0.00	-0.01*	-0.01	0.01*	0.00	0.00	00.00.00	0.01	0.00	0.01

R2	0.03	0.08	0.03	0.13	0.02	0.05	0.03	0.07	0.13	0.09

Adj. R2	0.03	0.08	0.03	0.13	0.02	0.05	0.03	0.07	0.13	0.09

* p < 0.05; ** p < 0.01

## Discussion

We reported results of a study of factors affecting participation and consequences of Italian GPs' participation in three forms of network organizations institutionalized in the INHS. We found that when GPs are able to choose the colleagues with whom to form a network, as in Italy, they tend to join networks of colleagues who are similar in age and have a similar number of patients in their rosters. We also found that participation in GP networks has a very weak effect on most of the performance indicators that were available. Most of the performance indicators do not seem to improve for GPs in networks nor are they more similar among GPs who are in the same network. An exception is for the number of prescriptions GPs print rather than handwrite, which is an important dimension of GP performance as it eases the burden of subsequent administrative work and it reduces misunderstandings of drug prescriptions and referrals. However, this may not be as fundamental as other dimensions more directly related to patient care. The evidence reported suggests that the GP networks of the type created and promoted in Italy at the end 1990s have had modest consequences on the ability of GPs to care for patients. Specifically, informal sharing of knowledge may not be present or it may not have an impact on the performance of GPs measured in most of the dimensions of performance.

This findings is consistent with those studies comparing in the UK context single handed practices to group practices showing that group practices do not yield better health outcomes than single handed practices [23,24].

Although every Italian LHA has autonomy to implement different organizational arrangements, the organization of GPs' activities it is fairly similar all over Italy as GPs are independent contractors whose relationship with LHAs is mainly regulated by the national contract. Therefore, our findings from the analysis of Piacenza LHA's data could likely be reproduced in other Italian LHAs which did not introduce different local-level arrangements.

Theoretical contributions on the network form of organization and on social networks allowed us to identify four characteristics of the GP network that are regulated by the Italian GPs' national contract which may have contributed to these results.

First, the contract focused on creating a structure for knowledge exchange but the presence of a structure does not imply that knowledge actually flows in the network. The creation of an interpersonal network does not suffice. Studies on social networks are often heralded as the tool to uncover the informal dynamics behind organizational charts [25]. However, because it is empirically difficult to gather data on actual flow of resources in networks, most empirical studies have focused on network structure assuming it is a good proxy for the flow of resources. Many scholars have also posited that structure has consequences on its own; in management the work of Ronald Burt [11,26,27] typifies this approach. A concern with structure can also be found in most public management reforms in Italy as organizational structure is considered the more objective part of the organization, it is therefore the one bureaucracies are most comfortable with. But the experience of GP networks in Italy suggests that the creation of a network structure through the concession of monetary incentives to "get together" and join a collaboration initiative does not guarantee the sharing of resources that rhetoric on network promises.

Second, the redundancy of the connections both in terms of redundancy of ties and of similarity of knowledge possessed by the people connected by those ties reduces the probability that new knowledge enters the network. Italian GP networks are essentially uni-professional organizational forms and, therefore, the diversity of knowledge resources is not leveraged. Moreover, the spontaneous birth of social networks is widely believed to be influenced by homophily (i.e., the tendency to create relationships with someone similar on relevant respects) [28]. The presence of homophily is confirmed in this setting as suggested by Table 4. GPs investigated in this study tend to join networks of colleagues who graduated from medical school in the same period. As they are also likely to have a similar education and knowledge, there is redundancy in the type of information GPs are able to access and thus there is a modest impact on professional relations.

Third, all network forms analysed here are organizational forms in which parts are undifferentiated. Each GP in the network performs the same type of activities on patients who in most cases always see the same GP. Therefore GP networks in Italy do not leverage the advantages of specialization that network forms of organization may create. Furthermore, the lack of strong interdependencies reduces the possibility of knowledge exchanges through observation, as can happen if GPs systematically see patients that are also seen by their colleagues.

Finally, the relationship between the INHS and its GPs has always mainly focused on monetary compensation with little attention paid to other forms of remuneration and incentives. The attitude of the INHS towards GPs has always been transactional rather than relational. A relational psychological contract is a work relationship that is understood by the employee and the employer not only as monetary but also as long-term or open-ended employment arrangements based upon mutual trust and loyalty [29]. A transactional attitude, typically reflected in contracts and explicit rules, may induce GPs to perceive their obligations as simply fulfilling the clear expectations of the organizational standards. A more relational approach may induce GPs to perceive their obligations as working together to achieve the complex goals for which GP networks were introduced and to invest in the relation per se.

The introduction of GP networks has focused on incentives to motivate GPs to enter such networks, without sufficient attention to the context necessary to allow networking to have an impact on the way professionals work. The government succeeded in having GPs accepting to join these collaborative initiatives, but the evidence we collected does not support the claim that these networks modify how professionals take care of their patients. Most likely, they are structural arrangements that do not change the pattern of communication among members.

## Conclusions

These findings suggest that managerial strategies which try to increase networking among employees should not focus solely on the creation of structure, as it has often been done in the INHS, but should focus instead on the creation of a need to access others' resources. Italian GPs in the same network are not interdependent, and therefore, being part of the same network implies neither interaction nor exchange. Moreover, in order to take advantage of the informational advantages of network form of organizations, members should be selected in order to have heterogeneous competencies so that each actor can find the knowledge needed in a specific situation by activating the more expert colleague in the network on the issue at stake. Another important lesson that can be learned from the introduction of GP networks in Italy is in the way the adoption of complex organizational structures should be promoted among GPs.

Focusing only on monetary incentives induces GPs to comply with the contractual requirement which, although superficial, is observable and thus enforceable. Since it is too difficult to enforce a contract requiring knowledge exchange among GPs, and it is also difficult to design an incentive structure to reward successful knowledge exchange in medicine, the type of exchange should be more relational in nature.

## Competing interests

The authors declare that they have no competing interests.

## Authors' contributions

DS: conceived the study, performed the statistical analysis, and participated in its design and coordination. GF conceived the study, and participated in its design and coordination. All authors read and approved the final manuscript.

## Pre-publication history

The pre-publication history for this paper can be accessed here:

http://www.biomedcentral.com/1472-6963/10/118/prepub
